# Supplementary material for: Multi‐dataset identification of innovative feature genes and molecular mechanisms in keratoconus
Source: J Cell Mol Med. 2024 Sep 19;28(18):e70079. doi: 10.1111/jcmm.70079 (PMC11412914; doi:10.1111/jcmm.70079)
Supplement: Supplementary file 2 — Material S2. [file JCMM-28-e70079-s002.zip › 7.GSEA analysis/5.RNF217-AS1/KEGG_MELANOMA.html]

Details for gene set KEGG\_MELANOMA[GSEA]

|  || Dataset | RNF217-AS1.group.cls#h\_versus\_l |
| Phenotype | group.cls#h\_versus\_l |
| Upregulated in class | l |
| GeneSet | KEGG\_MELANOMA |
| Enrichment Score (ES) | -0.37611532 |
| Normalized Enrichment Score (NES) | -1.2880931 |
| Nominal p-value | 0.12015504 |
| FDR q-value | 0.3971626 |
| FWER p-Value | 0.962 |
Table: GSEA Results Summary

  

Fig 1: Enrichment plot: KEGG\_MELANOMA      
 Profile of the Running ES Score & Positions of GeneSet Members on the Rank Ordered List

  

| SYMBOL | TITLE | RANK IN GENE LIST | RANK METRIC SCORE | RUNNING ES | CORE ENRICHMENT || 1 | PIK3R5 | na | 1058 | 0.434 | 0.0162 | No |
| 2 | FGF8 | na | 3265 | 0.280 | -0.0174 | No |
| 3 | FGF11 | na | 3425 | 0.274 | 0.0075 | No |
| 4 | FGF18 | na | 4467 | 0.235 | 0.0028 | No |
| 5 | FGF3 | na | 5480 | 0.200 | -0.0049 | No |
| 6 | BRAF | na | 5517 | 0.199 | 0.0155 | No |
| 7 | FGF5 | na | 6704 | 0.162 | -0.0013 | No |
| 8 | PIK3CD | na | 6778 | 0.159 | 0.0137 | No |
| 9 | PDGFB | na | 6818 | 0.158 | 0.0296 | No |
| 10 | IGF1R | na | 6917 | 0.155 | 0.0434 | No |
| 11 | E2F3 | na | 8526 | 0.115 | 0.0094 | No |
| 12 | FGF20 | na | 9057 | 0.104 | 0.0053 | No |
| 13 | FGF13 | na | 9302 | 0.099 | 0.0089 | No |
| 14 | FGF17 | na | 9838 | 0.089 | 0.0031 | No |
| 15 | FGF2 | na | 10549 | 0.077 | -0.0091 | No |
| 16 | PIK3CB | na | 11439 | 0.064 | -0.0279 | No |
| 17 | KRAS | na | 11445 | 0.064 | -0.0212 | No |
| 18 | CDH1 | na | 11891 | 0.057 | -0.0279 | No |
| 19 | PDGFA | na | 12021 | 0.055 | -0.0257 | No |
| 20 | IGF1 | na | 13325 | 0.038 | -0.0592 | No |
| 21 | MAPK1 | na | 13362 | 0.038 | -0.0562 | No |
| 22 | PDGFRB | na | 13387 | 0.038 | -0.0528 | No |
| 23 | EGF | na | 13646 | 0.034 | -0.0566 | No |
| 24 | PIK3CA | na | 14021 | 0.031 | -0.0641 | No |
| 25 | MAPK3 | na | 14127 | 0.029 | -0.0640 | No |
| 26 | HGF | na | 14364 | 0.026 | -0.0679 | No |
| 27 | MDM2 | na | 14406 | 0.026 | -0.0664 | No |
| 28 | FGF22 | na | 14720 | 0.022 | -0.0730 | No |
| 29 | FGF10 | na | 15522 | 0.014 | -0.0946 | No |
| 30 | FGF19 | na | 15680 | 0.012 | -0.0979 | No |
| 31 | AKT2 | na | 15816 | 0.011 | -0.1006 | No |
| 32 | HRAS | na | 15878 | 0.010 | -0.1013 | No |
| 33 | PIK3R1 | na | 16145 | 0.007 | -0.1082 | No |
| 34 | MAP2K2 | na | 16236 | 0.006 | -0.1101 | No |
| 35 | BAD | na | 16311 | 0.006 | -0.1116 | No |
| 36 | PDGFRA | na | 17252 | -0.004 | -0.1383 | No |
| 37 | NRAS | na | 17304 | -0.005 | -0.1393 | No |
| 38 | AKT1 | na | 18280 | -0.013 | -0.1660 | No |
| 39 | FGF14 | na | 18327 | -0.014 | -0.1658 | No |
| 40 | RB1 | na | 18364 | -0.014 | -0.1653 | No |
| 41 | CCND1 | na | 18820 | -0.019 | -0.1765 | No |
| 42 | RAF1 | na | 18969 | -0.020 | -0.1786 | No |
| 43 | FGF16 | na | 19735 | -0.027 | -0.1977 | No |
| 44 | PTEN | na | 19758 | -0.028 | -0.1954 | No |
| 45 | FGF6 | na | 20117 | -0.031 | -0.2024 | No |
| 46 | E2F2 | na | 20470 | -0.035 | -0.2088 | No |
| 47 | CDKN2A | na | 21184 | -0.042 | -0.2249 | No |
| 48 | MET | na | 22571 | -0.057 | -0.2588 | No |
| 49 | PIK3R2 | na | 22894 | -0.061 | -0.2615 | No |
| 50 | MAP2K1 | na | 23215 | -0.064 | -0.2639 | No |
| 51 | CDK6 | na | 23374 | -0.067 | -0.2613 | No |
| 52 | CDK4 | na | 24008 | -0.075 | -0.2714 | No |
| 53 | CDKN1A | na | 24177 | -0.078 | -0.2679 | No |
| 54 | EGFR | na | 24191 | -0.078 | -0.2599 | No |
| 55 | ARAF | na | 25299 | -0.095 | -0.2816 | No |
| 56 | FGF1 | na | 27173 | -0.130 | -0.3217 | No |
| 57 | PDGFC | na | 28424 | -0.160 | -0.3405 | No |
| 58 | FGF9 | na | 29191 | -0.181 | -0.3431 | No |
| 59 | PIK3CG | na | 30335 | -0.218 | -0.3527 | Yes |
| 60 | FGF23 | na | 31104 | -0.246 | -0.3484 | Yes |
| 61 | E2F1 | na | 31302 | -0.254 | -0.3268 | Yes |
| 62 | FGF7 | na | 31363 | -0.257 | -0.3009 | Yes |
| 63 | FGF12 | na | 31640 | -0.271 | -0.2797 | Yes |
| 64 | AKT3 | na | 31832 | -0.281 | -0.2549 | Yes |
| 65 | FGF21 | na | 32003 | -0.291 | -0.2285 | Yes |
| 66 | TP53 | na | 32227 | -0.304 | -0.2022 | Yes |
| 67 | FGF4 | na | 32529 | -0.327 | -0.1758 | Yes |
| 68 | PIK3R3 | na | 32895 | -0.352 | -0.1484 | Yes |
| 69 | FGFR1 | na | 34252 | -0.543 | -0.1292 | Yes |
| 70 | MITF | na | 34414 | -0.606 | -0.0687 | Yes |
| 71 | PDGFD | na | 34588 | -0.723 | 0.0040 | Yes |
Table: GSEA details [plain text format]

  

Fig 2: KEGG\_MELANOMA      
 Blue-Pink O' Gram in the Space of the Analyzed GeneSet

  

Fig 3: KEGG\_MELANOMA: Random ES distribution      
 Gene set null distribution of ES for **KEGG\_MELANOMA**
